# Fruiting phenology and nutrient content variation among sympatric figs and the ecological correlates

**DOI:** 10.1186/s40529-019-0275-9

**Published:** 2019-11-14

**Authors:** Yu-Ting Huang, Ya-Fu Lee, Yen-Min Kuo, Sing-Yi Chang, Chia-Ling Wu

**Affiliations:** Department of Life Sciences, National Cheng Kung University, 1 University Road, Tainan, 70101 Taiwan

**Keywords:** Dioecious, *Ficus*, Fruiting strategies, Monoecious, Nutrients, Phenology

## Abstract

**Background:**

Figs are key resources for tropical frugivores and display unique fruiting patterns. While monoecious figs support both seeds and wasp rearing, dioecious plants perform the tasks separately and produce seeded figs in smaller asynchronous crops. Thus dioecious females, compared to monoecious figs, may afford to invest more efforts to maximize seediness, or increase fruit pulp, water content, and nutrient rewards to attract frugivores for better seed dispersal. Yet size variation among and within fig species in either breeding system may lead to complicated resource allocation. We assessed fruiting phenology, measured fig morphological traits, and analyzed fig nutrient contents of the monoecious *Ficus caulocarpa* and *F. subpisocarpa* and the dioecious *F. ampelas* and *F. irisana* in a sympatric tropical forest to investigate species differences and size effects on fig functional traits and their ecological correlates.

**Results:**

All four species fruited nearly year-round. Monoecious figs’ inter-tree asynchronous crops had high peak mature crop sizes over much shorter fruiting periods than dioecious figs. Among trees, *F. subpisocarpa* and *F. irisana* were greater in fig-size and size variation, *F. caulocarpa* and *F. ampelas* comparatively displayed large variation in fig compositions. As fig size increased, water contents gradually increased in large-fig species, but seediness with a decreasing trend in small-fig species. Dioecious figs had lower pulp-seed ratio but tended to have higher water contents than monoecious figs, particularly within a similar size range. Dioecious figs also had higher carbohydrates, whereas monoecious figs contained higher fiber and lipid contents.

**Conclusions:**

Our study revealed species differences in certain fig functional traits that were correlated with fig size or their breeding systems, with substantial inter-tree variation. This partially supported the predictions regarding their fruiting strategies of aiding seed dispersal by frugivores, yet suggests a fruiting plasticity of individual trees subject to environmental constraints and their biotic interactions.

## Background

Seed dispersal by animals represents a widely noted interspecific interaction between consumers and their food plants (Howe and Smallwood 1982). While diffuse coevolution in plant-animal relationships has been well recognized for certain plants and their pollinators, e.g., yuccas and yucca moths (Powell 1992), dispersal syndromes have also been ascribed to certain frugivorous guilds (Corlett 2017; Herrera 1985), such as bird-dispersed versus bat- or primate-dispersed fruits (Gautier-Hion et al. 1985; Korine et al. 2000; Lomáscolo et al. 2008, 2010). Yet, seemingly mutualistic as it appears, both parties involved in seed dispersal are selected to gain self-interest. Thus as interesting as perceiving this relationship from the animal side (Sekercioglu 2006; Wunderle 1997), how plants may have evolved fruiting strategies via various functional traits to better attract frugivores is equally intriguing (Fleming and Estrada 1993; Howe and Smallwood 1982; Lord et al. 2002; Snow 1971).

Fruiting strategies of plants may be addressed in terms of size, morphology, and composition of fruits and seeds (Gautier-Hion et al. 1985; Izhaki 2002; Jordano 1995; Levey 1987; Mokotjomela et al. 2013; Schaefer et al. 2003; Snow 1981), crop size (Davidar and Morton 1986; Ortiz-Pulido and Rico-Gray 2000; Saracco et al. 2005; Wheelwright et al. 1984), and phenology (Milton et al. 1982; Poulin et al. 1999; Thompson and Willson 1979). Each strategy may be further divided into finer levels and be affected by biotic, i.e. diversity and foraging modes of major consumers (Eriksson and Ehrlén 1991; Thompson and Willson 1979; Wheelwright 1985), and abiotic factors like environmental conditions and seasonality (Lambert and Marshall 1991; Lotan and Izhaki 2013; Rathcke and Lacey 1985). This coping adaptation to dispersal agents is expected to be more obvious in pantropical regions where over 70% of plants produce fleshy fruits and over 90% of trees and shrubs depend on animal dispersers (Howe and Smallwood 1982; Levey et al. 2002).

*Ficus* (Moraceae) plants occupy a variety of habitats in pantropical areas and exhibit a wide range of life forms (Berg and Corner 2005; Harrison 2005). The extant 850 or so species constitute one of the largest flowering plant genera through complex life histories and different breeding systems, involving pollination aided by specialized fig-wasps and seed dispersal by diverse frugivores (Harrison and Shanahan 2005; Janzen 1979). Monoecious *Ficus* simultaneously support seed production and rearing fig-wasps, and syconia (hereafter referred to as figs for short) tend to mature synchronously in large crops over a short period, but may be asynchronously among plants so as to better sustain wasp populations (Harrison and Shanahan 2005). In contrast, gynodioecious (hereafter referred to as dioecious) species perform female and male tasks on separate plants. Functional males produce staminate flowers and gall flowers to provide pollens and wasp support, whereas females produce neuter flowers and seeded figs in generally smaller but longer-duration asynchronous crops (Herre et al. 2008). Continual fruiting tends to occur in environments suitable for seed dispersal and germination, and thus may overlap among trees in more-seasonal situations (Bain et al. 2014; Spencer et al. 1996).

Unlike monoecious figs, female dioecious figs are free from rearing fig-wasps, so may allocate more efforts toward seed production and subsequent dispersal (Herre et al. 2008; Lambert 1992). Asynchronous fruiting, smaller crops, and long crop periods also may allow dioecious *Ficus* to invest more in each fig fruit (Harrison and Yamamura 2003; Patel and Mckey 1998), such as increasing fruit size. With an increase in fruit size, figs may be selected by tradeoffs allocating resources to increase seediness, i.e. the number of potentially dispersible seeds per unit of fruit volume, or to pulp and compositional components to attract frugivores. In contrast, inter-plant asynchronous monoecious figs face tradeoffs allocating resources to crops that may inevitably encounter seasonal changes in fig-wasps, frugivores, or weather (Kjellberg and Maurice 1989; Bentos et al. 2014), and variation in fig investment may be substantial. The mechanisms regulating fig and crop sizes may also be related to fruit development and fruiting intervals, and the time available for accumulating energy reserves.

This study assessed the fruiting phenology, fruit composition, and nutrient contents of sympatric figs of both dioecious and monoecious species to examine interspecific variation in certain functional traits associated with the ecological correlates for avian seed dispersal. We tested the hypothesis that the breeding system and fruit size affect fig functional traits in coping with their seed dispersal by frugivores. Specifically, we predicted higher seediness in female dioecious figs than in monoecious figs. We also predicted higher water contents and attractive nutrients, but lower indigestible nutrients such as fiber and ash, in female dioecious figs. In either breeding system, larger-sized figs should contain higher water contents, pulp-seed ratios, and carbohydrates than smaller figs.

## Materials and methods

### Study area

The study was conducted in the Hengchun Tropical Botanical Garden (HTBG; 21° 960′–21° 962′ N, 120° 811′–120° 813′ E) within the Guijijaou Experimental Forest (450 ha in area, 200–300 m asl), at the southern edge of the Hengchun Peninsula, Taiwan. The region is mostly covered by primary forests rooted on uplifted coral reef karst terrain, representing one of the few remaining and the largest and least-disturbed lowland tropical monsoon forests on the island (Lee et al. 2008). Mean monthly temperatures above 20 °C in January and 26–28 °C in July–August and an annual rainfall of 2200–2300 mm typify its climate. Rainfall mainly occurs from April to October, particularly during the East Asian plum rain and typhoon seasons from May to September (Guijijaou Weather Station data, TFRI).

The HTBG contains primary and secondary forest fragments, small plantations of various types of native and some introduced plants, grassland patches, and managed facilities such as nursery plots and greenhouses in a mosaic pattern. Details of the vegetation composition are described elsewhere (Lee et al. 2008). In this area, monoecious figs include white bark figs *F. benjamina* L., large-leaved figs *F. caulocarpa* (Miq.) Miq., Chinese banyan *F. microcarpa* L. f. var. *microcarpa*., and fruit figs *F. subpisocarpa* Gagnep. Dioecious figs comprise King’s figs *F. ampelas* Burm. f., rough-leaved figs *F. irisana* Elm, angular-fruit figs *F. septica* Burm. f., and white figs *F. virgata* Reinw. *ex* Bl. (HTBG data, TFRI). They are all arboreal species but *F. septica* and *F. virgate* may also grow as shrubs and in smaller size (Tzeng 2004).

### Tree sampling and phenology

We first conducted pilot surveys of dispersion and abundance of local fig assemblages in the area, primarily among forest fragments. We then selected larger trees from the most common monoecious and female dioecious figs that were at least > 5 cm in diameter at breast height (Dbh), > 1.5 m high, contained previous fruiting records (YFL unpubl. data) and were accessible for monitoring and fruit sampling over the coral reef terrain. These sampled trees represent two distinct groups, *F. caulocarpa* (*n* = 13) and *F. subpisocarpa* (*n* = 11) of Subg. *Urostigma* (Gasp.) Miq., and *F. ampelas* (*n* = 9) and *F. irisana* (*n* = 8) in Subg. *Sycidium* (Miq.) Mildbr. & Burret. These species all produce typical orange-reddish or black-purple figs at the mature stage, which are positioned at leaf axils but those of *F. caulocarpa* and *F. subpisocarpa* also commonly at leafless branches (Tzeng 2004), and are fed frequently by frugivorous birds (Wu 2017; Walther et al. 2018).

From March 2013 to February 2015, fruiting phenology of monoecious figs and female dioecious figs was assessed for 4–5 days weekly by monitoring every crop of each tree, and fig phase and crop size recorded (Damstra et al. 1996; Kjellberg and Maurice 1989). We monitored the initiation and followed each fig phase (A: pre-female receptive, B: female receptive, C: inter-floral, D: wasp-dispersal, and E: post-floral fig dispersal; Galil and Eisikowitch 1968) and estimated the fig development period. This period covers phases A–D in monoecious figs but only phases A–C (lacking D) in dioecious figs. For overlapped fruiting in dioecious figs, we used the massive emergence of phase A to separate different crops. The total length of each crop period was monitored until the crop size was less than 5% of the initial E stage.

### Canopy and crop size

We estimated the canopy volume (CV, m^3^) by a modified formula for an approximate half sphere, CV = 2/3π × *r*^2^ × (canopy thickness), for each tree, where canopy thickness is measured as the distance between the top and bottom of the canopy (Thorne et al. 2002), where *r* is an averaged value of eight radius measurements from equally divided angles. We visually estimated crop sizes for trees with few or small amounts of larger-sized fruits (Chapman et al. 1992), but applied a stratified sampling method (Kalko et al. 1996) when fruits were too numerous to be counted reliably. For the latter, we divided branches into three classes and defined the largest fig-bearing branches as the 3rd class, which merged into the 2nd class and then further merged into the 1st class, often bifurcating directly from the main trunk. We randomly selected 30 tertiary branches to tally the number of figs on each branch, then obtained the mean value (*n*). The 1st class branches were counted (*b*_*1*_), then six 1st class branches were randomly selected to estimate the mean number of 2nd class branches per 1st class branch (*b*_*2*_) and the mean number of the 3rd class branches per 2nd class branch (*b*_*3*_). The crop size was then estimated as *n* × *b*_*1*_ × *b*_*2*_ × *b*_*3*_ (Kalko et al. 1996).

### Fig sampling, fig composition, and morphological measurements

We sampled fig fruits at the peak E stage from at least two crops each tree, without specifying the year or season factors due to various crop durations, between-crop intervals, and practical difficulties of clearly distinguishing the often continuous and overlapping crops in dioecious figs. In sampling, we randomly selected six fig-bearing twigs and collected all figs, and totally 11,308 figs were collected for analyses (*F. caulocarpa*, 5437; *F. subpisocarpa*, 3497; *F. ampelas*, 1772; *F. irisana*, 602). We measured the fig fresh mass (fm) using a PL303 digital balance (Mettler Toledo, Greifensee, Switzerland) and then kept them in cold preservation. In the laboratory, we randomly selected 100 figs, or all available figs if fewer than 100, from each sample, measured the length (*l*), width (*w*), and height (*h*) between the head and ostiolar, along three axes using CD-6″ BS digital calipers (Mitutoyo, Kanagawa, Japan). We estimated the fig volume (mm^3^) using a modified formula for a sphere as V = (4/3)π × (1/2)^3^(*l* × *w* × *h*). Figs were oven-dried at 50 °C, then the dry mass (dm) was measured, and the water content (%) was calculated as WC = [(fm – dm)/fm] × 100. We randomly examined 30 mature figs under a Stemi DV4 microscope (Zeiss, Jena, Germany) to obtain the seediness and pulp-seed ratio in mass (Izhaki 2002).

### Nutrient contents

We used the fig samples collected for morphological and compositional measurements to analyze nutrient contents of pulp only, with seeds and galls excluded. For each crop sample, it took at least 45 g of dry pulp for a complete set of analysis of lipids, protein, carbohydrates, crude fiber, ash, and calcium contents, following the AOAC’s (1995) protocols. We estimated lipids by a Soxhelt extraction, and protein by using the Kjeldahl method where nitrogen was converted to crude protein by a factor of 6.25 (Weiblen et al. 2010). Fiber content was obtained by boiling 2 g of pulp samples in neutral detergent to rinse away the soluble fraction and extract cell wall components including cellulose, hemicellulose, and lignin that cannot be used or digested by most plant-eating animals. We used an ash furnace (JA-150, JorFai, Hsinchu, Taiwan) set to 550 °C to estimate the ash content in 5 g of samples (Conklin and Wrangham 1994). After ashing, samples were dissolved in 3 N hydrochloric acid (37.5%) and diluted to 100 ml with water for the mineral analysis. We also used a nitrogen-free extract to indicate available carbohydrates that were digestible, such as starches and sugars. This was obtained by subtracting the proportions of lipid, protein, fiber, and ash contents from 100% (Herrera 1982b; Sanmee et al. 2003). Each ash sample was repetitively tested 10 times for absorbance values of the wave length of 422.7 nm using atomic absorption spectroscopy (SensAA, GBC-903, Hampshire, IL, USA) for estimating the concentration of calcium. They were compared to absorbance values obtained from a standard Ca-solution at a concentration of 998 ± 2 mg/L (Merck, Kenilworth, NJ, USA) in quantities of 2–10 ppm with an increment of 2 ppm (Rode et al. 2003) to estimate the calcium contents.

### Data analysis

Data are presented as the mean ± standard errors (SE) unless otherwise noted, and were analyzed using SPSS Statistics 17.0 (Chicago, IL, USA), with the significance level set to *α* = 0.05. Data were logarithmic transformed as necessary to meet the requirements of normality and homoscedasticity, or analyzed using alternative statistics when appropriate (Zar 2010). The latter applied when examining the peak crop size at maturation and the development, maturation, and total fruiting durations among species using a Kruskal–Wallis test, followed by a posterior Dunn-test to locate interspecific differences. We conducted correlation analyses to examine the relationships between DBH, tree height, and canopy volume with crop size, fig volume, and seed amount for each species. We performed MANOVA followed by Tukey’s honest significant difference (HSD) test to examine differences in tree variables (DBH, canopy volume, and height) among species, and numbers of fruiting trees per month by incorporating weekly observations. We examined fig volume among species by ANOVA, and used a general linear model (GLM) of MANCOVA analysis with fig size as a covariate to examine species and fig-size effects on seediness, pulp-seed ratio, and water content of figs. Nutrient contents in mean values were presented for figs using each tree as the final source of variance. A principal component analysis (PCA) was applied to identify major trends in nutrient content variation, with major factor loadings set to > 0.6 (Schaefer et al. 2003).

## Results

### Fruiting phenology

**Fig. 1:**
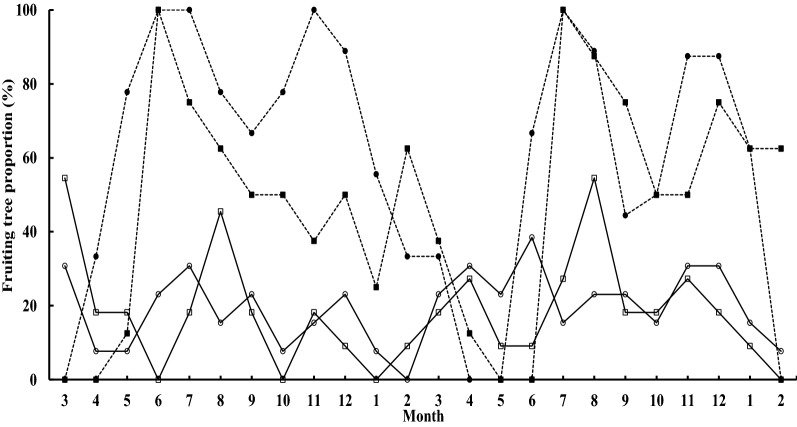
Fruiting phenology of monoecious (line with open circle: *F. caulocarpa*; line with open square: *F. subpisocarpa*) and dioecious (line with filled circle: *F. ampelas*; line with filled square: *F. irisana*) figs by respective fruiting-tree percentage in the Hengchun Tropical Botanical Garden (HTBC), Kenting

The figs fruited nearly year-round, with all four species in 14 months and three of the four species in 22 months bore mature fruits over the study period. Fruiting generally peaked in midsummer and secondarily in early winter when all sampled trees were bearing fruits (Fig. 1). *Ficus caulocarpa* ceased fruiting only in February; that of *F. subpisocarpa* occurred in January, June, and October, whereas the fruiting of the two dioecious figs declined more similarly from January to April–May. On average, more dioecious figs (ANOVA, *F*_(3, 92)_ = 9.78, *p* < 0.001; *F*. *ampelas*: 5.3 ± 0.7, 59.7 ± 7.1%, Tukey HSD, *p* < 0.001; *F. irisana* 3.8 ± 0.5, 47.4 ± 6.4%, *p* < 0.05) were fruiting each month than were monoecious *F. caulocarpa* (2.5 ± 0.3, 19.6 ± 2.0%) and *F. subpisocarpa* (2.0 ± 0.3, 18.6 ± 3.1%).

*Ficus caulocarpa* showed major interspecific differences in tree variables, being greater in DBH, canopy volume, and tree height than the other species (Pillai’s trace *V* = 0.8, *F*_(9, 108)_ = 4.36, *p* < 0.001; Table 1). Although tree variables across species were generally correlated to each other (DBH vs. canopy volume, *r* = 0.86, *p* < 0.05; canopy volume vs. tree height, *r* = 0.59, *p* < 0.05), we found a slight positive correlation only between DBH and crop size (*r* = 0.57, *p* < 0.05), but no significant relationships between either DBH, tree height or canopy volume with crop size, fig volume, or seed number in *F*. *caulocarpa*. No correlations were found between any tree or fig variables for the other three species either (all *p* values > 0.05).

**Table 1:** Mean (± SE) DBH, canopy volume, and tree height of four species of figs assessed in the Hengchun Tropical Botanical Garden (HTBG), Kenting, and the comparison examined by MANOVA

Species	DBH (cm)	Canopy volume (m^3^)	Tree height (m)
*F. caulocarpa*^1^	162.95 ± 20.72^a***^	1098.47 ± 213.51^a***^	11.13 ± 0.58^a**^
*F. subpisocarpa*^1^	36.63 ± 4.16^a^	197.46 ± 47.92^a^	9.90 ± 0.75^b*^
*F. ampelas*^2^	45.29 ± 9.0^a^	173.84 ± 31.07^a^	7.33 ± 0.31^ab^
*F. irisana*^2^	31.0 ± 6.08^a^	159.82 ± 66.14^a^	8.23 ± 1.0^a^

A species trait value followed by a letter and asterisks indicates a significant difference from values of other species with the same letter but without an asterisk under the same trait variable, examined by post hoc comparisons following the main test

^1^ Monoecious

^2^ Dioecious

^*^p < 0.05, ^**^p < 0.01, ^***^p < 0.001

Figs also differed in the mean peak crop size as entering fruit maturity (Kruskal–Wallis test, *H* = 99.57, *d.f.* = 3, *p* < 0.001; Table 2). *Ficus caulocarpa* bore a larger crop size than *F. subpisocarpa* (Dunn test, *Q* = 10.27, *p* < 0.05) and the dioecious *F. ampelas* (*Q* = 12.49, *p* < 0.05) and *F. irisana* (*Q* = 24.27, *p* < 0.05), and *F. irisana* produced the smallest crop size (*Q* > 11.4, *p* < 0.05). While monoecious and dioecious figs’ developmental durations were alike (*H* = 4.21, *d.f.* = 3, *p* = 0.24), they differed in mature (*H* = 96.90, *d.f.* = 3, *p* < 0.001) and total fruiting periods (*H* = 74.62, *d.f.* = 3, *p* < 0.001; Table 2). Maturation of monoecious figs was significantly shorter than that of dioecious figs (Dunn test, all *Q* values > 8.9, *p* < 0.05), and no species difference was found within either breeding system (*Q* values < 1.2, *p* > 0.05). This resulted in a pattern of the total fruiting periods similar to that of maturation where monoecious figs were shorter than dioecious figs (*Q* values > 8.3, *p* < 0.05), with no species differences within either breeding system (*Q* values < 1.08, *p* > 0.05; Table 2).

**Table 2:** Mean (± SE) crop size and fig development, maturation, and total fruiting durations per crop-tree of four species of figs (*n* = tree number, crop number) assessed in HTBG and the comparison examined using Kruskal–Wallis tests

Species	Crop size	Duration (days)		
		Development	Maturation	Fruiting
*F. caulocarpa* (13, 27)	113,283.5 ± 21,481.6^a*^	33.8 ± 1.6	15.3 ± 1.5^a^	48.8 ± 2.2^a^
*F. subpisocarpa* (11, 25)	13,057.3 ± 2536.0^a,b*^	31.5 ± 2.7	12.2 ± 0.9^a^	44.1 ± 3.0^a^
*F. ampelas* (9, 43)	18,280.1 ± 3543.0^a,b*^	32.8 ± 4.2	59.0 ± 4.5^a*^	92.9 ± 6.4^a*^
*F. irisana* (8, 37)	2096.9 ± 579.3^ab^	38.3 ± 3.3	63.1 ± 4.5^a*^	100.9 ± 5.1^a*^

A species trait value followed by a letter and asterisks indicates a significant difference from values of other species with the same letter but without an asterisk under the same trait variable, examined by post hoc comparisons following the main test

^*^p < 0.05

### Fig size and internal composition

**Fig. 2:**
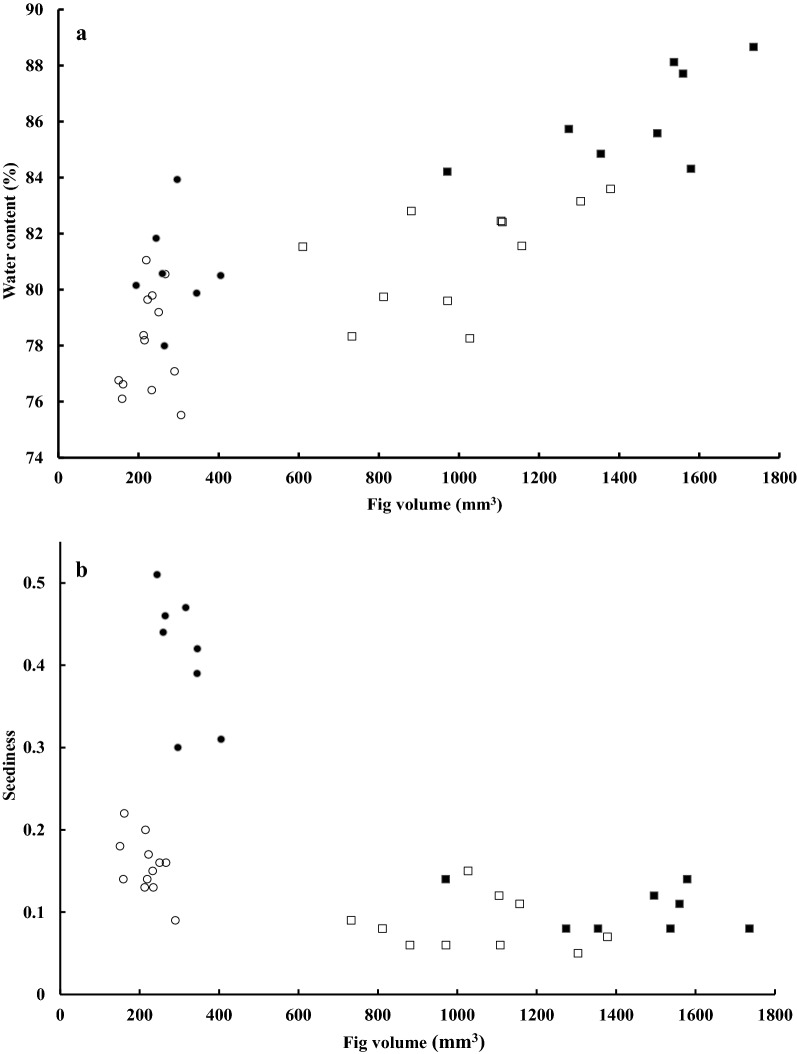
The correlation between **a** water contents, and **b** seediness, with fig volume sizes in monoecious (open circle: *F. caulocarpa*; open square: *F. subpisocarpa*) and dioecious (filled circle: *F. ampelas*; filled square: *F.irisana*) species assessed in HTBC, Kenting

The syconium size differed among the four figs (ANOVA, *F*_(3, 37)_ = 170.04, *p* < 0.001) and also between any two species in paired comparisons (Tukey’s HSD, all *p* values < 0.05). *Ficus irisana* topped among the four species, and was followed by *F. subpisocarpa*, and then smaller *F. ampelas* and *F. caulocarpa* (Table 3). By controlling fig size as a covariate, the fig breeding system appeared to affect seediness, the pulp-seed ratio, and water content of syconia (MANCOVA, *V* = 1.38, *F*_(9, 108)_ = 10.22, *p* < 0.001). Dioecious figs were higher in seediness (*F. ampelas*) and water contents (*F. ampelas* and *F. irisana*), but lower in pulp-seed ratios (*F. ampelas* and *F. irisana*; Table 3). Across breeding systems, fig size also affected interspecific differences in certain fig traits (*V* = 0.39, *F*_(3, 34)_ = 7.23, *p* < 0.001). As fig size increased, water contents gradually increased in large-size figs (*F. subpisocarpa*, *r* = 0.55, *p* < 0.05; *F. irisana, r *=0.66, *p* < 0.05) but with more variation in *F. caulocarpa* (*r* = 0.15, *p* < 0.05) and *F. ampelas* (*r* = 0.23, *p* < 0.05), whereas a decreasing trend in seediness occurred in small-fig species (*F. caulocarpa*, *r* = 0.56, *p* < 0.05; *F. ampelas, r *=0.64, *p* < 0.05) but less apparent in *F. subpisocarpa* (*r* = 0.11, *p* < 0.05) and *F. irisana* (*r *=0.30, *p* < 0.05; Fig. 2).

**Table 3:** Mean (± SE) fig volume (mm^3^), seediness (seeds/mm^3^ fig volume), pulp-seed ratios, and water contents (%) of four species of figs (*n* = tree number) assessed in HTBG and the comparison examined by MANCOVA

Species	Volume	Seediness	Pulp-seed ratio	Water content
Fcau (13)	224.78 ± 13.29^a,b^	0.17 ± 0.01^a,b**^	8.36 ± 0.82^a*^	78.10 ± 0.51^a,b,c^
Fsub (11)	1008.06 ± 71.15^a,b*^	0.08 ± 0.01^a,b^	14.13 ± 3.74^a***b***^	81.22 ± 0.58^a,c*^
Famp (9)	296.74 ± 21.27^a,b^	0.39 ± 0.03^a***^	3.05 ± 1.34^a^	80.14 ± 0.65^a,b*^
Firi (8)	1438.39 ± 83.27^a*^	0.10 ± 0.01^a^	4.67 ± 0.63^b^	86.15 ± 0.63^a*^

A species trait value followed by a letter and asterisks indicates a significant difference from values of other species with the same letter but without an asterisk under the same trait variable, examined by post hoc comparisons following the main test

Fcau: *F. caulocarpa*, Fsub: *F. subpisocarpa*, Famp: *F. ampelas*, Firi: *F. irisana*

^*^p < 0.05, ^**^p < 0.01, ^***^p < 0.001

### Nutrient contents

**Fig. 3:**
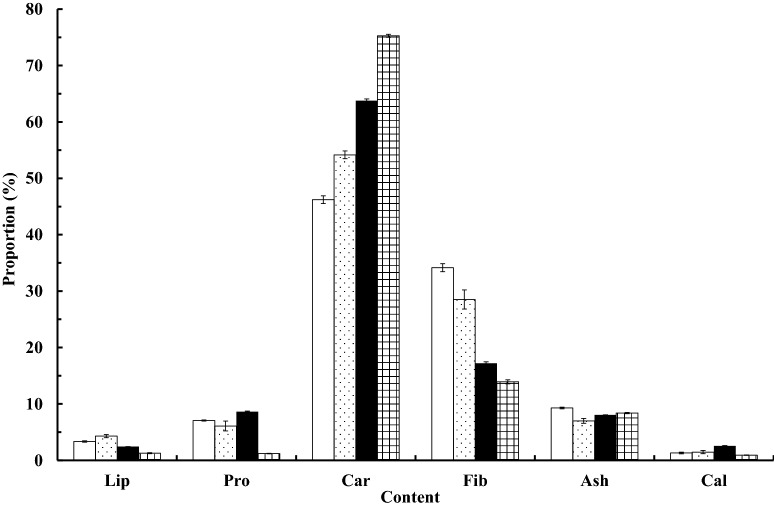
Mean (± SE) nutrient contents (%) of lipids, proteins, carbohydrates, fiber, ash, and calcium in monoecious (open squaree: *F. caulocarpa*;  : *F. subpisocarpa*) and dioecious (filled square: *F. ampelas*;  : *F. irisana*) figs assessed in HTBC, Kenting

Nutrient contents of figs were largely similar, with carbohydrates dominating the organic nutrients, followed by protein, but proportions of crude fibers and ash relative to organic nutrients varied as did those of dietary minerals such as calcium. Within either breeding system, larger-sized syconia (i.e., dioecious *F. irisana* and monoecious *F. subpisocarpa*) contained higher carbohydrates but lower crude fiber than did smaller-sized syconia of *F. caulocarpa* and *F. ampelas* (Fig. 3).

**Fig. 4:**
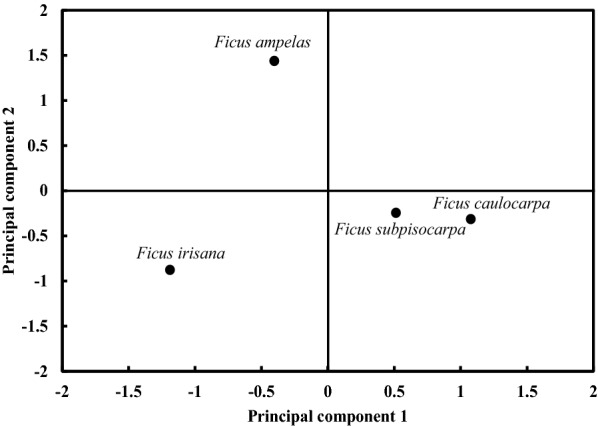
Principal component analysis of nutrient contents of four fig species assessed in HTBC (see Table 4 for each nutrient component). PC1, with increasing lipid and fiber but decreasing carbohydrate contents, and PC2, with increasing calcium and protein contents, accounted for 80.65% of the total variation

Carbohydrates, crude fiber, and lipids accounted for more than 53% of the variance along PC1, with carbohydrates negatively correlated with the other two nutrient contents. In PC2, calcium and protein were responsible for more than 27% of the variance and were positively correlated with each other (Table 4). PC1 and PC2 together explained 80.65% of the variance. Along PC1, monoecious *F. caulocarpa* and *F. subpisocarpa* were higher in crude fiber and lipids, but lower in carbohydrates, than the dioecious *F. ampelas* and *F. irisana*. Along PC2, however, *F. ampelas* had the highest and *F. irisana* the lowest calcium and protein contents, whereas the two monoecious species were alike in the middle (Fig. 4).

**Table 4:** Component loadings and variance proportions explained in a principal component analysis for nutrient contents of four species of figs assessed in HTBC

Nutrient content^a^	PC1	PC2
Lipids	0.900	^b^
Proteins	0.599	0.801
Carbohydrates	− 0.998	^b^
Fiber	0.983	^b^
Calcium	^b^	0.966
Variance (%)	53.267	27.378

^a^ Ash showed no correlation in either component and was excluded

^b^ Loadings < 0.25

## Discussion

Organisms are often selected for balance between investing in the size and number of offspring under energy constraints (Stearns 1992). In plants, large seeds may sustain harsh conditions longer or be more competitive for space and resources than small seeds, whereas small seeds are transported over long distances easily and may bear a chance of germinating in suitable less-crowded habitats (Bentos et al. 2014; Parciak 2002). Fig syconia generally contain numerous tiny seeds, but variation occurs in fruit size and also other traits such as the crop size and phenology between the breeding modes (Harrison and Shanahan 2005; Kjellberg and Maurice 1989). Monoecious figs usually produce large synchronous crops, but may exhibit intra-tree asynchronous fruiting in highly seasonal environments (Hossaert-McKey and Bronstein 2001; Lin et al. 2008). Dioecious species also may display phenological variation under various climatic and environmental conditions (Bain et al. 2014).

In our study, monoecious *F. caulocarpa* and dioecious *F. irisana* represent the two extremes where the former produces the smallest figs, the largest crop size, and the least seed number per fig, whereas the latter displaying the opposite. We did not measure actual seed size of each fig; if assuming a similar seed size for the two species, ca. 0.25–0.26 mm^3^ (Tzeng 2004), which would generate the highest seed investment per crop in *F. caulocarpa* and the lowest in *F. irisana* at a difference of almost 16 times. This is consistent with the much shorter crop period and less crops in *F. caulocarpa* than in *F. irisana*. In the area we studied, *F. caulocarpa* was represented by large-sized trees that typically also contributed to large crop sizes. Large synchronous crops may attract more diverse frugivorous dispersers of infrequent occurrence, which may increase the chance of fig seeds being dispersed to suitable habitats farther away and result in the common pattern of a wider distribution (Harrison 2003).

Tree sizes appeared to confound with the breeding system in affecting crop sizes; yet, the tree size-crop size relationship was not consistent in the rest species. *F*. *caulocarpa* and *F. subpisocapa* fruited asynchronously among trees throughout most of the year, with a low fruiting proportion each month. This concurred with findings in northern Taiwan and may aid maintaining the wasp populations (Bain et al. 2015). In contrast, dioecious *F. ampelas* and *F. irisana* showed more apparent seasonality with inter-plant synchrony, which is consistent with studies conducted at other sites (Bain et al. 2014; Tzeng 2004). The intra-tree asynchronous fruiting in dioecious figs, however, requires a longer period of investment in fruits and may result in a sexual fruiting dichotomy, where female figs’ fruiting period is longer while that of males are closer to monoecious figs (Patel and McKey 1998).

Female figs are free from the constraint of wasp-rearing, thus are expected to maximize their seed numbers (Harrison and Yamamura 2003) or attractiveness to frugivores (Dumont 2004; Lambert 1992; Patel and McKey 1998; Weiblen et al. 2010), both may enhance seed dispersal. Our result suggested that female dioecious figs generally contained higher water contents and seediness than monoecious figs and is consistent with that prediction. Dioecious figs had lower pulp-seed ratios; this supports a previous finding of a smaller increase in pulp than in seeds among species (Herrera 1987) because of energy constraints. Dioecious figs may achieve this by increasing the water content, and large-sized figs containing higher water contents supports the positive physiological relationship between fruit size and water (Lotan and Izhaki 2013). It also concurs with the notion that as the fig size increases, a higher proportion of water is needed to aid transpiration and lowering of the temperature, so as to avoid wasp deaths due to overheating in figs (Patiño et al. 1994).

Nevertheless, variation occurred in fig size between species in either breeding system, which in turn may cause differnces in fig traits among species (Suleman et al. 2013). All four species, particularly small-sized figs, showed a more apparent decreasing trend in seediness as the fig size increased, which may be related to their interactions with fig-wasps. As the fig size increases, so does available ovules for pollination and subsequent seeds (Herre 1996). An insufficient number of fig-wasps may cause insufficient pollination in dioecious figs and in turn a smaller increase in seeds compared to fig size; whereas in monoecious figs, overabundant fig-wasps may result in an over exploitation of ovules by wasps and a lowered seed number (Nefdt and Compton 1996). In addition, as the fig size increases, the seed size instead of the seed number may increase as a consequence (Bentos et al. 2014). For instance, the seed size of *F. subpisocarpa* was larger than that of *F. caulocarpa* (Tzeng 2004), and suggests the need to include seed size in future studies.

Among PC1 variables, dioecious figs tended to contain higher carbohydrates, but lower lipids and crude fiber, than monoecious figs, which may be associated with their high water content (Debussche et al. 1987; Izhaki et al. 2002). Carbohydrates have a lower energy supply than lipids, but are absorbed more quickly (del Rio et al. 1992), thus should be preferred by most frugivores. In contrast, lipids are not easily absorbable, particularly for birds with rapid food passage through their digestive tracts (Fuentes 1994). Yet, high lipids in foods may aid to acquiring and restoring energy by wintering birds and migrants in winter times (Herrera 1982a). Although we only measured nutrient contents of two figs in either breeding system, other sympatric monoecious figs in our study area like *F. benjamina* and *F. microcarpa* were reported to have similar carbohydrate (48.5% and 53.0%) and lipid contents (3.5% and 4.0%; Corlett 1996). Females of all 12 dioecious species studied in New Guinea also indicated higher soluble carbohydrate but lower fiber contents than their conspecific functional males that were closer to most monoecious figs reported (Weiblen et al. 2010). These are consistent with our findings and provide support for the nutritional differences between monoecious and dioecious figs.

The lipid contents in these figs, however, were much lower than those of some other fruits commonly fed on by birds, such as *Macaranga tanarius* (L.) Muell.-Arg. (29%, Corlett 1996), *Leea guineensis* G. Don (20%, Curtis 2004), and *Trema orientalis* (L.) Bl. (45%, Bollen et al. 2004), thus may not convey a significant effect on food choice of wintering birds. Monoecious figs also often contain higher fiber proportions, almost twice that of dioecious figs, which are indigestible to animals (Weiblen et al. 2010), offering lower energy returns and possibly appearing less attractive to frugivores. Within either breeding system, large-sized *F. caulocapra* and *F. irisana* contained higher carbohydrates, but lower fiber and protein. This concurs with our explanation regarding the water content’s effect on proportions of hydrophilic and hydrophobic nutrients (Izhaki et al. 2002). From frugivores’ viewpoint, large-sized figs with a small crop may still attract frugivores by the higher water and carbohydrate contents. In contrast, higher fiber and protein contents in smaller-sized figs would generate satiation more quickly (Rodrigo et al. 2012), which prevents fruits from being overly consumed by the same birds on single or few trees over a lengthy time, thus may better contribute to seed dispersal. This can be particularly beneficial to monoecious figs that fruit synchronously and usually in large crops.

Protein, ash, and calcium contents appeared not different between monoecious and dioecious figs; instead they showed variations within each system. Both proteins and calcium were highest in *F. ampelas* but lowest in *F. irisana* along PC2. The calcium content found in *F. ampelas* (2.55%) was even higher than some of the highest ever recorded, including those from Belize (1.91%), Uganda (1.52%), and Indonesia (1.21%; O’Brien et al. 1998). The high calcium and protein contents suggest *F. ampelas* an attractive food resource, particularly in breeding seasons, which requires further confirmation (YFL, unpubl. data). In contrast, the highest proportion of ash occurred in *F. caulocarpa*, but the lowest in *F. subpisocarpa*. The fact that species of higher ash contents did not necessarily contain high calcium contents suggests other minerals may have affected ash contents, such as potassium. We did not analyze this element, so cannot verify this speculation, but Wendeln et al. (2000) indicates that potassium can reach a considerable amount in some figs.

## Conclusions

Our study, given constrained by a limited species pool, indicates species differences in certain functional traits that are correlated with fig size or the breeding system. Our findings convey insights into understanding how the differences in fig traits are associated with fruiting strategies that may aid fig removal and seed dispersal by frugivores, and offer implications for ecological dynamics of fig assemblages in tropical-subtropical areas. In addition, small-sized figs showed larger individual variations in certain traits over fig-size range. Both demand and call for further studies of a more expanded species pool to better illuminate fruiting plasticity of figs and their interactions with pollinators and frugivores.

## Data Availability

The datasets for this study are available from the corresponding author on personal request.
